# Irisin: Still chasing shadows

**DOI:** 10.1016/j.molmet.2020.01.016

**Published:** 2020-01-31

**Authors:** Elke Albrecht, Lisa Schering, Friedrich Buck, Konrad Vlach, Hans-Christof Schober, Christian A. Drevon, Steffen Maak

**Affiliations:** 1 Institute of Muscle Biology and Growth, Leibniz Institute for Farm Animal Biology (FBN), Dummerstorf, Germany; 2 Institute of Clinical Chemistry, University Medical Center Hamburg-Eppendorf, Hamburg, Germany; 3 Department of Internal Medicine I, Municipal Hospital Suedstadt Rostock, Rostock, Germany; 4 Department of Nutrition, Institute of Basic Medical Sciences, University of Oslo, Oslo, Norway

**Keywords:** FNDC5, Skeletal muscle, Adipose tissue, Plasma, Transcript, Mass spectrometry

## Abstract

**Objective:**

Considerable uncertainty remains regarding the veracity of measuring myokine irisin more than seven years after its original description. Unresolved issues include the nature of transcription of the irisin precursor fibronectin type III domain containing 5 (FNDC5) gene across species, the reliability of irisin levels measured with commercial enzyme-linked immunosorbent assays (ELISAs), and the overall validity of the recently published reference values for human serum measured with quantitative mass spectrometry. We utilized multiple species and measures to evaluate the robustness of commonly used reagents and methods for reporting irisin.

**Methods:**

Amplification of cDNA was used to assess the FNDC5 transcript patterns in humans and mice. The specificity and sensitivity of different irisin antibodies were examined via western blotting. Quantification of circulating native irisin was conducted with mass spectrometry using an absolute quantification peptide for irisin.

**Results:**

We show that there is a greater transcript diversity of human FNDC5 than currently annotated, but no indication of the expression of transcripts leading to a truncated form of irisin. Available irisin antibodies still bind to patterns of unspecific serum proteins, which compromise reliable measurements of irisin with ELISAs. Absolute quantification of irisin with labeled peptides by mass spectrometry is an advanced method but requires a multi-step sample preparation introducing uncontrollable variations in the measurement.

**Conclusion:**

Our data represent an explicit warning against measuring circulating irisin using available methods. Measuring irisin is akin to chasing shadows.

## Highlights

• Transcription pattern of the host FNDC5 gene is not conserved from mouse to human.
• Irisin antibodies detect neither circulating irisin nor FNDC5 in humans and mice.
• Sample preparation impairs exact quantification of irisin by mass spectrometry.
• Results on irisin levels in humans and mice are still unreliable.

## 1 Introduction

The putative myokine irisin has been the subject of debate since its description in 2012 by Boström et al. [1]. The major controversy involved the suitability of the antibody used for the first detection, the existence of full-length irisin in humans due to a non-canonical start codon in the gene encoding its precursor fibronectin type III domain containing 5 (FNDC5), the reliability of commercial enzyme-linked immunosorbent assays (ELISAs) for irisin measurements, and the physiological role of irisin in humans [[2], [3], [4], [5], [6]]. The scientists who discovered irisin addressed some of these points of contention [7]. They reported detection of glycosylated as well as deglycosylated native irisin in human plasma samples using western blotting with a commercial antibody. They also used quantitative mass spectroscopy to measure circulating irisin in sedentary and trained individuals. The authors concluded that irisin exists, circulates, and is inducible by exercise in humans. Although the study involved only a few individuals with a marginal increase in irisin, some considered the data sufficient to settle the debate [8]. Others suggested independently reproducing these results [9].

In a recent study, integrins, primarily complexes involving alpha V integrin, were identified as long sought receptors mediating the effects of irisin on bone and fat in mice [10]. Furthermore, the effects of irisin on bone remodeling and induction of a thermogenic program in white adipose tissue was demonstrated at much lower concentrations than previously described. Lourenco et al. [11] reported that irisin rescued synaptic plasticity and memory in murine Alzheimer's models.

Despite these results on physiological effects of irisin in mice, debate continues regarding the molecular weight (MW) range in which irisin (predicted MW: 12.7 kDa) is expected on western blotting of biological samples. Our previous study found that non-glycosylated bacterial irisin was ∼13 kDa on gels and glycosylated irisin from HEK293 cells had an apparent MW of 20–25 kDa [6]. Full-length FNDC5, without its signal peptide, has a predicted MW of ∼20 kDa. Glycosylation should result in an increase in the MW to ∼27 kDa. However, several studies reported that the MWs of irisin and FNDC5 strongly deviated from predictions in mice and humans ([1,[11], [12], [13], [14]]). Moreover, irisin levels measured with different commercial ELISAs were reported in a range from picograms to micrograms per milliliter of serum or plasma ([[15], [16], [17], [18], [19], [20], [21], [22]]).

Due to the uncertainty of irisin measurements and transcription, we aimed to identify FNDC5 transcripts in human muscle at the mRNA and protein levels, update the reliability of irisin antibodies, and detect and quantify irisin in different species using mass spectrometry.

## 2 Materials and methods

### 2.1 Ethics approval

Human serum samples were obtained from the MyoGlu human exercise study [5,23]. The study adhered to the Declaration of Helsinki and was approved by the National Regional Committee for Medical and Health Research Ethics, North Tromsø, Oslo, Norway. The study was registered with the US National Library of Medicine Clinical Trials registry (NCT01803568). Written informed consent was obtained from all of the participants prior to any study-related procedures. A description of the samples used in this study is provided in Supplemental Table S1.

Human muscle and subcutaneous fat samples were obtained from a study at the Municipal Hospital Suedstadt, Rostock, Germany. This study was approved by the Ethics Commission of the Medical Faculty at the University of Rostock in accordance with national law and the ICH-GCP guidelines (registry number A 2013-152). All of the participants provided prior written informed consent. As part of the informed consent process, the participants’ rights as research subjects, exceptions to confidentiality, and the possible risks involved were clearly explained.

### 2.2 Animal serum, plasma, muscle, and fat samples

Serum samples for species comparison were either purchased from commercial suppliers (donkey, goat, rabbit, rat, and baboon), or used from previous studies of different species [6,[24], [25], [26]]. The FNDC5 gene of all of the analyzed species possessed a canonical start codon in contrast to humans. This could theoretically lead to a more efficient transcription of the gene. The sequence of the irisin peptide within the FNDC5 gene was 100% identical in all of the species including humans.

Muscle and fat samples of calves were obtained from an ongoing investigation that was approved by the Animal Protection Board of the Leibniz Institute for Farm Animal Biology and the Animal Care Committee of Mecklenburg-Western Pomerania, Germany (State Office for Agriculture, Food Safety, and Fisheries; LALLF M-V/TSD/7221.3-1-052/15). All of the animal samples represent the species with no further characterization.

### 2.3 Transcript analyses of human and murine FNDC5

Human and mouse skeletal muscle tissue was homogenized with Xiril Dispomix (Xiril, Hombrechtikon, Switzerland) and then incubated in QIAzol lysis reagent (Qiagen, Hilden, Germany) according to the provided protocol. The RNA was isolated and purified with NucleoSpin Extract II reagent (Macherey–Nagel, Duren, Germany) as described by the manufacturer. Total RNA quantification was conducted using a NanoDrop ND-1000 spectrophotometer (Peqlab, Erlangen, Germany) and the RNA integrity was determined with an Experion Automated Electrophoresis System using an RNA StdSens analysis chip (Bio-Rad, Munich, Germany). Then 100 ng of total RNA was added to a 20 μL reaction volume using an iScript cDNASynthesis Kit with a blend of oligo (dT) and random hexamer primers (Bio-Rad) for first strand cDNA synthesis as described by the manufacturer. The transcript expression was qualitatively analyzed by standard PCR with the primer combinations listed in Supplementary Table S2. Briefly, amplification of the target sequences took place in a 10 μL reaction volume with 5 ng cDNA, 2 μmol/L primer pairs, and PCR master mix (2×) (Thermo Fisher Scientific, Bonn, Germany) in a pecSTAR 96 universal thermocycler (Peqlab). The amplification started with an initial denaturation at 94 °C for 4 min, followed by 35 cycles at 94 °C for 30 s, a template-specific annealing temperature (Supplementary Table S2) for 1 min, 72 °C for 1 min, and the final step at 72 °C for 7 min. The PCR products were resolved on 3.0% agarose gels containing Roti-GelStain (Carl Roth GmbH, Karlsruhe, Germany) and visualized under UV light (Quantum, Peqlab). Amplicons were purified with a High Pure PCR Product Purification Kit (Roche Diagnostics, Mannheim, Germany), and the PCR products were sequenced by cycle sequencing with PCR primers and analyzed on a 3500 Genetic Analyzer (Applied Biosystems/HITACHI, Darmstadt, Germany). The sequences were compared with reference sequences using a CLC Main Workbench (v. 8.1, CLCbio, Aarhus, Denmark).

### 2.4 Antibodies and recombinant proteins

Four polyclonal irisin antibodies (designated as A, B, C, and D) and one polyclonal FNDC5 antibody (E) were used in this study. Details on the antibodies are provided in the upper part of Table 1. Recombinant irisin, expressed in *E. coli C41*, and recombinant glycosylated irisin expressed in HEK293 cells was a generous gift of Dr. Harold P. Erickson (Duke University, Durham, NC, USA). Details on the properties and production were previously described by Albrecht et al. [6]. Both irisin forms were used as size references and positive controls for serum/plasma samples in western blotting.

**Table 1:** Antibodies used to detect irisin in this study and other research.

#	Manufacturer/vendor	Cat. #	Lot #	Immunogen	Lab^a^/[Ref.]		Observed bands in plasma/serum	Remarks
**Antibodies used in this study with previous results**								
A	Phoenix	G-067-29	01701–1	Recombinant irisin	FBN		See results	
B	Erickson Lab^b^	n/a	n/a	Recombinant irisin	FBN		See results	
C	BioVision	6635	2A126635	Recombinant irisin	FBN		See results	
D	AdipoGen	AG-25B-0027	A25481312/A27881602	Recombinant irisin	FBN Bos [7]		See results 25/12 kDa (native/+neb^c^), human	50 μg protein
					Bos [14]		22 kDa (+NEB), mouse	80–100 μg protein
E	Abgent	AP8746b	SA121121BK	FNDC5 C-terminus	FBN		n/a; see results	Used in tissue only
**Antibodies used for irisin detection in other studies**								
	Abcam	Ab93373	Not specified	FNDC5 C-terminus	Bos [1]	32/20 kDa (native/+PNG^d^), human, mouse		Irisin peptide is not covered by this immunogen
					Bet [12]	32/24 kDa (native/+PNG), human		
					Tai [39]	22 kDa (+PNG), human		
	Phoenix	Not specified	Not specified	Recombinant irisin	Cho [13]	12 kDa (native), mouse		20 μL serum
	Phoenix	EK-067-52	Not specified	Recombinant irisin	Lei [37]	17 kDa (+NEB), human		Ab from ELISA kit
	BioTrend	A000170-01	01478/01483	Recombinant irisin	FBN [26]	15/12 kDa (native/+PNG), mouse		Ab discontinued
	Cell Sig Techn	Proprietary	N/A	unknown	Bos [10]	20 kDa (native), mouse		

a **FBN**: Leibniz Institute for Farm Animal Biology (FBN) Dummerstorf, Germany; **Bos**: Harvard University Medical School, Boston, MA, USA; **Bet**: National Institutes of Health, Bethesda, MD, USA; **Tai**: Chang Gung University College of Medicine, Taoyuan, Taiwan; **Cho**: Third Military Medical University, Chongqing, P.R. China; **Lei**: Center for Pediatric Research, University of Leipzig, Germany.

b Produced in the laboratory of Dr. H. P. Erickson (Duke University, Durham, NC, USA).

c Native = untreated serum; +NEB = samples deglycosylated with protein deglycosylation mix (NEB).

d +PNG = samples deglycosylated with PNGase F.

### 2.5 Protein preparation and western immunoblotting

Protein from muscle and adipose tissue was extracted using CelLytic MT lysis buffer (Sigma–Aldrich, Taufkirchen, Germany) supplemented with protease inhibitor cocktail (P8340, Sigma–Aldrich) as previously described [26,27]. Plasma and serum samples were albumin-depleted (Aurum Affi-Gel Blue mini columns, Bio-Rad) or albumin- and IgG-depleted (ProteoExtract Albumin IgG Removal Kit, Calbiochem, Merck Millipore, Darmstadt, Germany) following the manufacturer's instructions. The samples were concentrated using AmiconUltra-2 Centrifugal Filter Devices (Merck Millipore). Recombinant non-glycosylated irisin was diluted into albumin-depleted bovine plasma to final concentrations of 4, 2, 1, 0.5, 0.25, 0.125, 0.0625, and 0.031 ng/mL and 2 μL of each were separated on gels to estimate the detection range of the tested antibodies. The recombinant glycosylated irisin, serum/plasma, and muscle samples were deglycosylated either with PNGase F or Protein Deglycosylation Mix II (New England Biolabs, NEB, Frankfurt, Germany) according to the manufacturer's instructions. All of the proteins were mixed with 2x/4x Laemmli buffer including 10% β-mercaptoethanol, denatured for 5 min at 95 °C, and separated with polyacrylamide gel electrophoresis (PAGE) using Criterion TGX 12% gels (Bio-Rad) or 12% SDS-PAGE mini gels. Two different molecular weight markers (PageRuler, Triple Color Protein Standard III, Serva, Heidelberg, Germany) were used to determine the molecular weights of the observed protein bands. The proteins were transferred to polyvinylidene difluoride (PVDF) membranes (Tans-Blot Turbo Transfer Pack, Bio-Rad) with a semi-dry blotter (Trans-Blot, Bio-Rad). The membranes were stained with Coomassie Blue to verify the equal loading of the gels and proper transfer of the proteins. After blocking for 1 h using Roti-Block (Roth), the membranes were incubated with primary antibodies (Table 1) overnight at 4 °C at the dilution recommended by the manufacturer. The membranes were washed four times and subsequently incubated with horseradish peroxidase-conjugated secondary antibody (rabbit IgG TrueBlot, 1:25,000; 18-8,816, eBioscience, Frankfurt, Germany). Highly sensitive chemiluminescence substrate (Super Signal West Femto, Thermo Fisher Scientific) was used to detect the antibody binding. Chemiluminescence was recorded with a Chemocam HR-16 imager (INTAS, Göttingen, Germany) and quantified USING LabImage 1D software (Kapelan Bio-Imaging, Leipzig, Germany). The specificity of the primary antibody binding was tested using parallel blots incubated with antibody blocked with recombinant irisin or FNDC5 blocking peptide, respectively, prior to incubation.

### 2.6 Mass spectrometric measurements

#### 2.6.1 Sample preparation

Gels were stained with colloidal Quick Coomassie and the potentially irisin-containing bands were removed. The proteins were reduced with DTT (10 mmol/L, 56 °C, 30 min), the cysteine residues modified with iodoacetamide (55 mM, ambient temperature, 20 min in the dark), and the in-gel protein digested with trypsin (8 ng trypsin/μL; sequencing grade modified trypsin, Serva) in 50 mmol/L NH_4_HCO_3_, 37 °C, 16 h). After digestion, the gel pieces were repeatedly extracted (50% acetonitrile/5% formic acid) and the combined extracts were dried down in a vacuum concentrator and re-dissolved in 20 μL 0.1% formic acid.

The isotopically labeled synthetic peptide FIQEVNTTTR (^13^C-labeled at Val-5, Peptides & Elephants, Potsdam, Germany) was dissolved in 0.1% formic acid/15% acetonitrile to produce a stock solution of 500 ng/μL.

#### 2.6.2 LC/MS conditions

The LC/MS/MS runs were conducted on Orbitrap Exactive and Orbitrap Fusion mass spectrometers (Thermo Fisher Scientific, Bremen, Germany) connected to an Acquity nanoUPLC (Waters, Eschborn, Germany). Two μL of each sample were loaded onto a reversed-phase (RP) trapping column (Symmetry C18 Trap Column; 100 Å, 5 μm, 180 μm × 20 mm) and washed with 1% buffer B for 5 min. The peptides were eluted onto a RP capillary column (nanoAcquity Peptide BEH analytical column; 130 Å, 1.7 µm, 75 μm × 200 mm, buffer A: 0.1% formic acid in H_2_O; buffer B: 0.1% formic acid in acetonitrile) and separated under the following conditions: 10 min at 3% solvent B followed by a gradient from 3% to 35% buffer B for 35 min (flow: 250 nL/min). Eluting peptides were ionized by ESI in the positive mode using a fused silica emitter (I.D. 10 μm, New Objective, Woburn, MA, USA) at a capillary voltage of 1,800 V.

#### 2.6.3 DDA measurements

For peptide identification, mass spectrometric measurements were conducted in the data dependent acquisition (DDA) mode on an Orbitrap Fusion instrument. Survey scans were recorded in the Orbitrap (resolution 120,000, AGC target 2 × 105, maximum injection time 50 ms, scan range 300–1,500 *m*/*z*, profile data). To acquire the fragment spectra, precursor selection was done in a quadrupole (isolation window 1.6 *m*/*z*) and fragment detection in the instrument's ion trap (AGC target 1 × 10^4^, maximum injection time 35 ms, HCD fragmentation at 30%, scan range 100–1,200 *m*/*z*, centroid data).

Data were searched with Proteome Discoverer 2.0 software (Thermo Fisher Scientific) using the following parameters: trypsin (full) cleavage, missed cleavages: 2, precursor mass tolerance: 10 ppm, fragment mass tolerance: 0.6 Da, dynamic modification: oxidation at Met, static modification: carbamidomethylation at Cys, target FDR for PSMs: 0.01, target FDR for peptides: 0.01, data banks searched: Swiss-Prot (UniProt release 2017_1) human or rat complemented with the irisin sequence (AsN at the glycosylation sites replaced by Asp), and common contaminants (e.g. keratins).

#### 2.6.4 PRM measurements

For measurements in the parallel reaction monitoring (PRM) mode [28], MS spectra were acquired on the Orbitrap Exactive mass spectrometer with a resolution of 70,000 FWHM at *m*/*z* 200 (AGC target 1 × 10^6^, maximum injection time 240 ms, scan range 400–1,200 *m*/*z*, profile data). The MS/MS spectra were obtained by HCD fragmentation (normalized collision energy 25, 27, and 29) with a resolution of 17,500 FWHM (AGC target 2 × 10^5^, max. IT 50 ms, isolation window 2.0 *m*/*z*, scan range 80–1,250 *m*/*z*, centroid data). The PRM measurements were conducted under conditions similar to those described by Jedrychowski et al. [7]. The data were quantified using Skyline version 4.2 [29].

## 3 Results

### 3.1 High FNDC5 transcript diversity in human skeletal muscle

**Figure 1:**
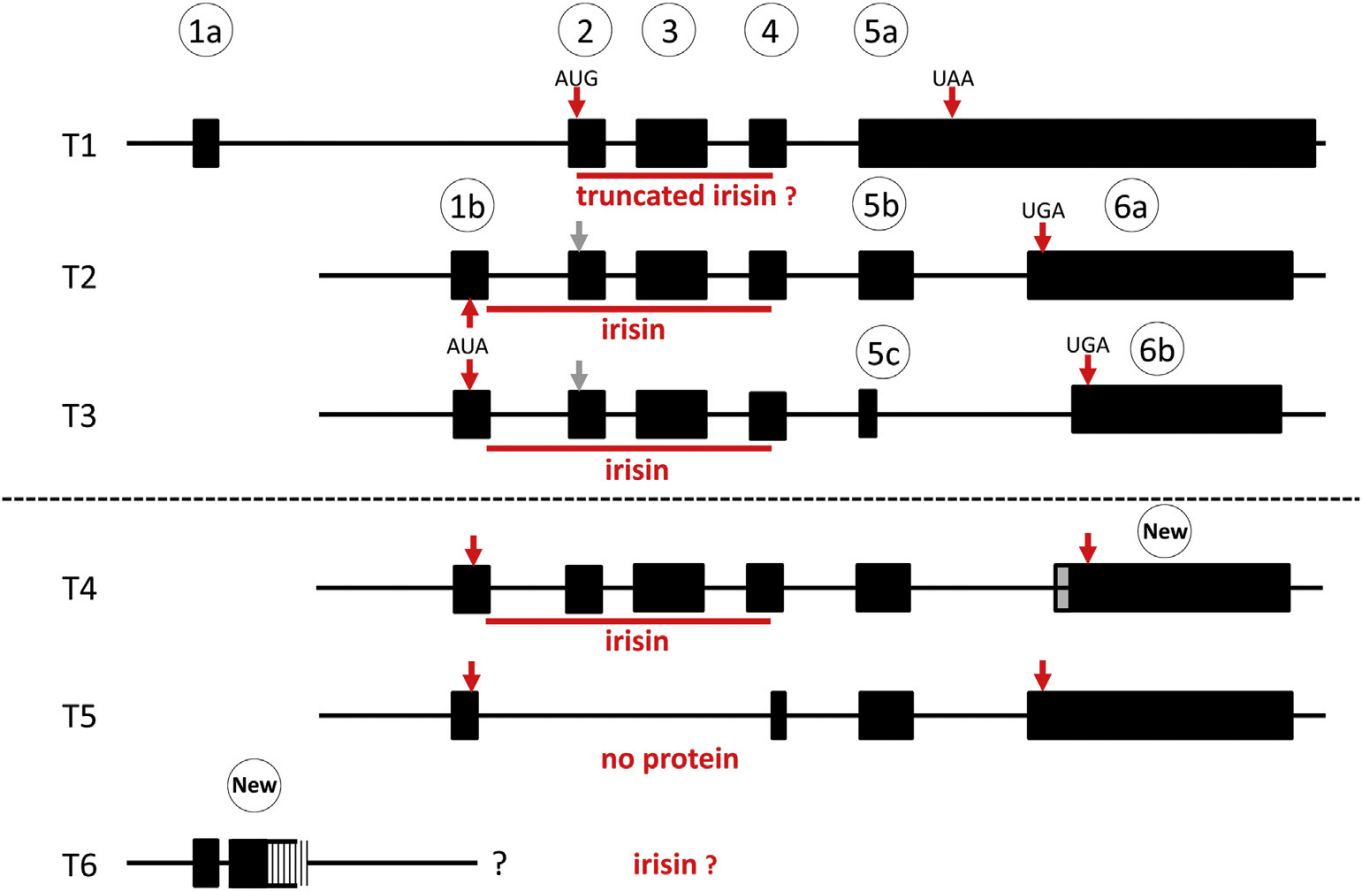
**Annotated and expressed FNDC5 transcripts in human skeletal muscle.** T1-T3: Transcripts annotated in GenBank. T1: NM_001171941.2. T2: NM_153756.2. T3: NM_001171940.1. T4-T6: Additional transcripts expressed in human skeletal muscle in this study. Exon numbering (circles) refers to data published by Albrecht et al. [6]. The GenBank-derived exons correspond to the following exons in Ensembl Release 98 (09/19): 1a: ENSE00001811973. 1b: ENSE00001862258. 2: ENSE00001751279. 3: ENSE00003591295. 4: ENSE00003704806. Annotation of exons 5a-6b differs between GenBank and Ensembl.

There is currently no clear evidence of the transcription of human FNDC5 from either a canonical or non-canonical start codon and the results of the amount and length of the respective products. To clarify this, we first amplified three fragments allegedly representative of three FNDC5 transcripts annotated in GenBank (NM_001171941.3, NM_153756.3, and NM_001171940.2; T1-3 in Figure 1) in human skeletal muscle cDNA with primers described by Kim et al. [30]. The C_p_ values indicated robust expression of all three transcripts at similar levels (data not shown). However, sequencing the amplicons revealed that the primers for transcript T3 co-amplified a hitherto unknown fragment herein called T4 (Figure 1). Furthermore, the fragments apparently representative of the annotated transcripts 1–3 do not consider the 5’ exon structure of human FNDC5. To obtain this information, we tried to amplify further fragments of transcripts T1-T3 and searched for respective expressed sequence tags (ESTs) in NCBI databases supporting the structure of the identified transcripts.

Amplification of the T1, T2 and T3 annotated transcripts with all exons failed in our muscle samples although 12 different primer combinations were tested (Supplementary Figure S1 and Supplementary Table S2). However, our results provide evidence that one or more transcripts covering the complete irisin sequence are expressed in human muscle (Supplementary Figure S1). Additionally, we observed a truncated fragment that was sequenced and is shown as T5 in Figure 1. Additional database searches revealed numerous ESTs comprising exon combinations deviating from the T2 and T3 annotated transcripts. The coding sequences of these aberrant transcripts were changed only in the C-terminal region and did not affect the irisin sequence.

The annotated T1 of human FNDC5 comprises an alternative first exon that contains neither a canonical nor a non-canonical start codon. The resulting protein is translated only from a downstream canonical AUG codon (Figure 1) and thus lacks the signal peptide and the first 44 amino acids of the proposed irisin peptide. This specific transcript is supported only by a single EST from a neuroblastoma cell line (GenBank BU151187.1) and was not found in our investigation. One EST (CV815003.1) links exon 1a with a downstream genomic fragment. We successfully amplified this fragment (T6 in Figure1). All attempts to link this “new” exon to further downstream exons (1b, 2, 3, or 4; Figure 1) by cDNA-PCR were not successful, indicating that neither the annotated transcript T1 nor an unknown T1-related FNDC5 transcript are expressed in human skeletal muscle. This makes transcripts encoding truncated irisin peptides unlikely.

In contrast, the sequencing of murine FNDC5 mRNA revealed only a single transcript in the skeletal muscle identical to reference sequence NM_027402.4 (data not shown). Amplification of a further predicted transcript (XM_006503212.3) failed.

### 3.2 FNDC5 protein is detected in human, bovine, and murine muscle but only in murine fat

Expression of the irisin precursor FNDC5 was analyzed in human, murine, and bovine skeletal muscle and adipose tissue with an antibody against the C-terminus (antibody E; Table 1).

**Figure 2:**
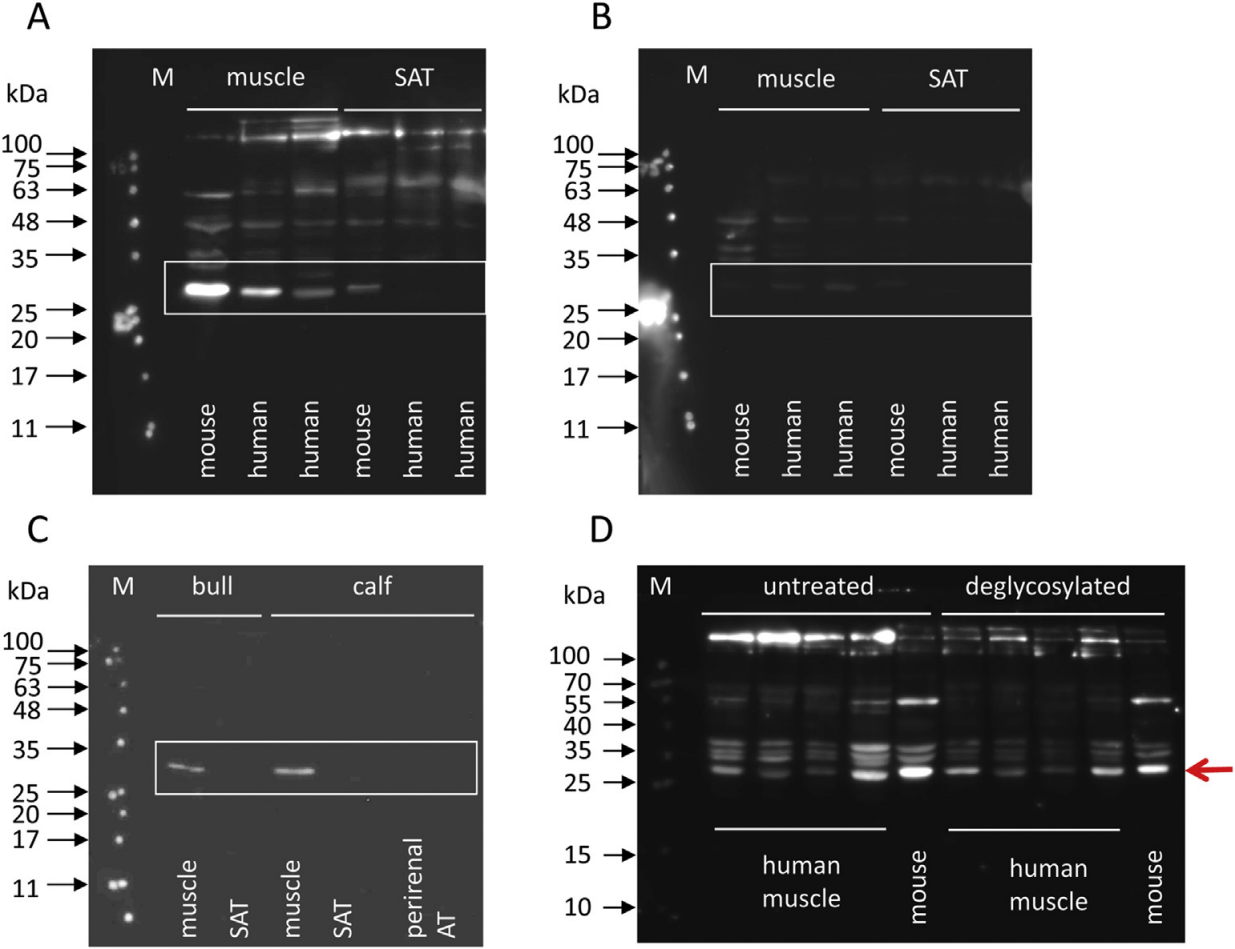
**Detection of FNDC5 in different species with antibody E. A:** FNDC5 in human and mouse muscle and adipose tissue. **B:** The antibody was pre-incubated with a blocking peptide in a parallel blot. **C:** FNDC5 in bovine muscle and adipose tissues. **D:** FNDC5 in four human and one mouse muscle sample before and after deglycosylation. M: molecular weight marker. SAT: subcutaneous adipose tissue. AT: adipose tissue. Regions of interest are marked by boxes or a red arrow.

This antibody targets the transmembrane and cytoplasmic region of FNDC5, which is identical in the investigated species. A specific band was obtained in the skeletal muscle of humans, mice, calves, and adult cattle. In contrast, FNDC5 was detected only in murine subcutaneous adipose tissue (SAT) but not in human and bovine SAT (Figure 2A–C). Brown adipose tissue (perirenal fat) of two calves did also not reveal an immunoreactive band for FNDC5 (Figure 2C). The observed MW of FNDC5 in muscle (∼27 kDa) corresponds to expectations. A band of truncated FNDC5 from the hypothetical transcript T1 (Figure 1) was not observed in the human muscle samples in concordance with our analysis of human FNDC5 transcripts.

Specificity of the binding was confirmed by a strongly quenched signal when the antibody was pre-incubated with a blocking peptide prior to incubation of the blot (Figure 2B).

Repeating this experiment revealed a different band pattern, although the 27 kDa band was observed again (red arrow in Figure 2D). However, apparently unspecific staining was observed in contrast to the previous experiment with the same antibody C (Figure 2A). The samples were deglycosylated with the Protein Deglycosylation Mix II (NEB; Figure 2D) because FNDC5 harbors two N-glycosylation sites. Surprisingly, no band shifts were observed after deglycosylation (Figure 2D). Thus, the bands from the human and mouse muscle suspected to be specific for FNDC5 based on our previous experiment (red arrow) were subjected to mass spectrometric analysis (see Section 3.6).

### 3.3 Sensitivity and specificity of irisin antibodies

**Figure 3:**
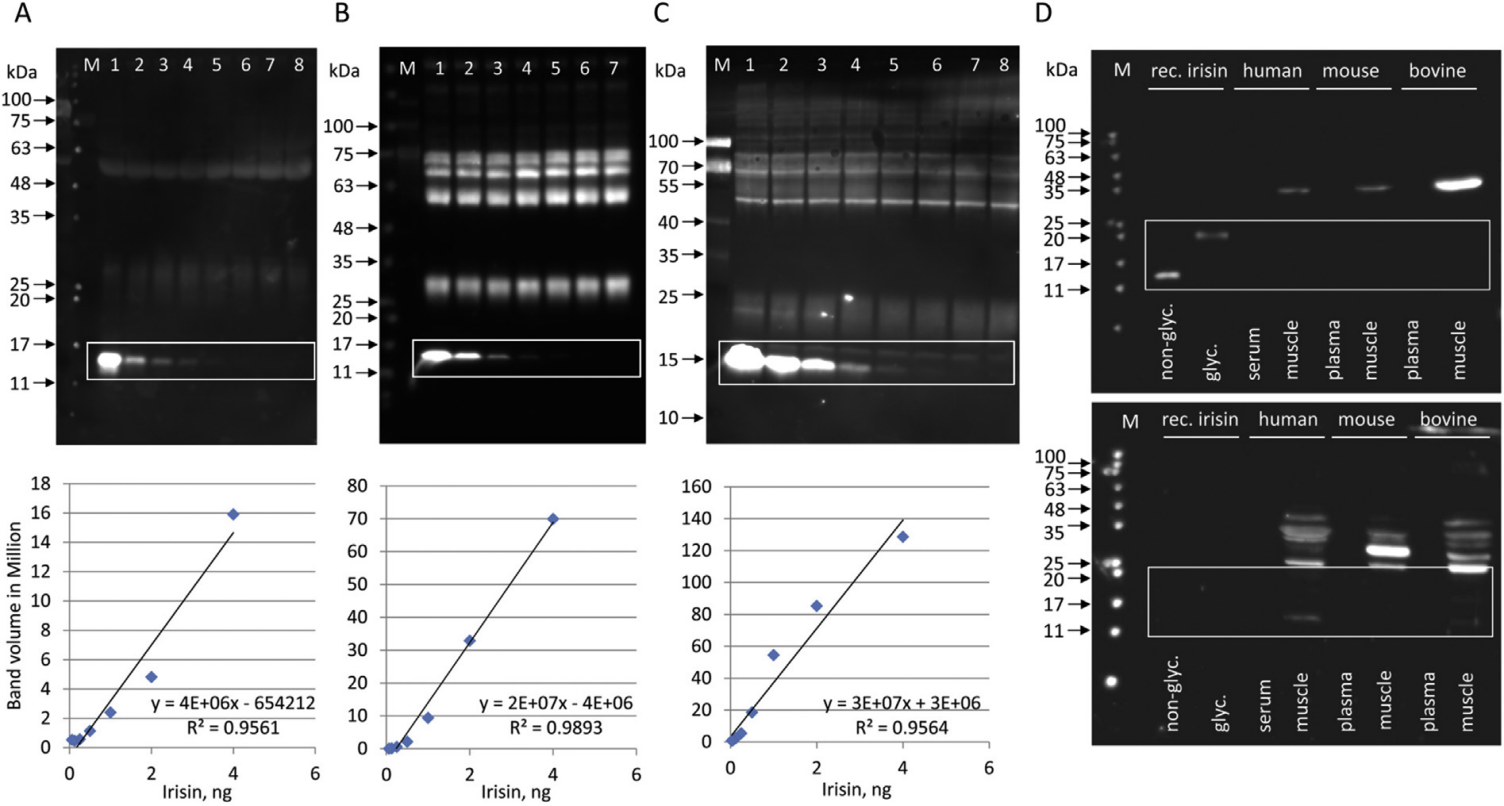
**Sensitivity and specificity of irisin antibodies. A-C:** Detection of different amounts of recombinant irisin with antibodies A, B, and C. **A:** Irisin amounts per lanes 1–8: 20, 4, 2, 1, 0.5, 0.25, 0.125, and 0.062 ng. The first lane, containing 20 ng recombinant irisin, was excluded to calculate the linearity (when included: R^2^ = 0.9781). **B:** Irisin amounts per lane 1–7: 4, 2, 1, 0.5, 0.25, 0.125, and 0.062 ng. **C:** Irisin amounts per lane 1–8: 4, 2, 1, 0.5, 0.25, 0.125, 0.062, and 0.031 ng. Linearity of detection is shown for each antibody in the lower panel (details in Supplementary Figs. S2A–C). **D:** Detection of non-glycosylated and glycosylated recombinant irisin, and FNDC5/irisin in human*, mouse and bovine serum/plasma, and muscle with antibody C (upper panel). The antibody was pre-incubated with recombinant irisin in a parallel blot (lower panel). ^∗^Human serum sample: HS 1 (acute exercise; details in Supplementary Table S1). M: molecular weight marker. Regions of interest are marked by boxes.

We determined the sensitivity of antibodies against recombinant bacterial irisin using western blotting. The recombinant protein was diluted in albumin-depleted bovine plasma as a biological matrix with no detectable native irisin [25]. Antibody A (Figure 3A, Table 1) was used in a commercial ELISA (Phoenix Pharmaceuticals, EK-067-29, Mannheim, Germany). Antibody B (Figure 3B) was raised against full-length bacterial recombinant irisin (provided by Dr. Harold P. Erickson, Duke University, Durham, NC, USA), and antibody C (Figure 3C) was provided by BioVision (Milpitas, CA, USA). All of the antibodies showed comparable performances with linearity in a range of 4 ng–0.125 ng recombinant irisin per lane, which marked the limit for reliable detection of irisin in western blotting as revealed by image analysis (Figure 3A–C, Supplementary Figures. S2A–C). Unspecific binding was observed for all three antibodies with different intensities and patterns.

Irisin antibody C was further tested in serum and muscle (Figure 3D). Recombinant irisin prior to and after deglycosylation was used as a positive control and 20 μg of total protein from deglycosylated human, murine and bovine serum or plasma, and muscle of all three species were analyzed. As shown in the upper panel of Figure 3D, specific signals for both forms of recombinant irisin were observed but not for native irisin in any of the plasma/serum samples. A single band at ∼30–35 kDa was found in the muscle protein of all three species, considerably larger than the expected MW for glycosylated FNDC5 (27 kDa). Blocking the antibody by prior incubation with recombinant protein caused complete suppression of the specific signals for recombinant irisin forms but resulted in multiple unspecific bands in the muscle samples of all three species (Figure 3D lower panel). The tested irisin antibody did not reliably bind to full-length FNDC5 although it was directed against the central part of this protein. Thus, the identity of the 30–35 kDa band stained in muscle remains unclear but is unlikely to be FNDC5.

### 3.4 Deglycosylation of recombinant irisin with PNGase F or a deglycosylation enzyme mix is similarly efficient

**Figure 4:**
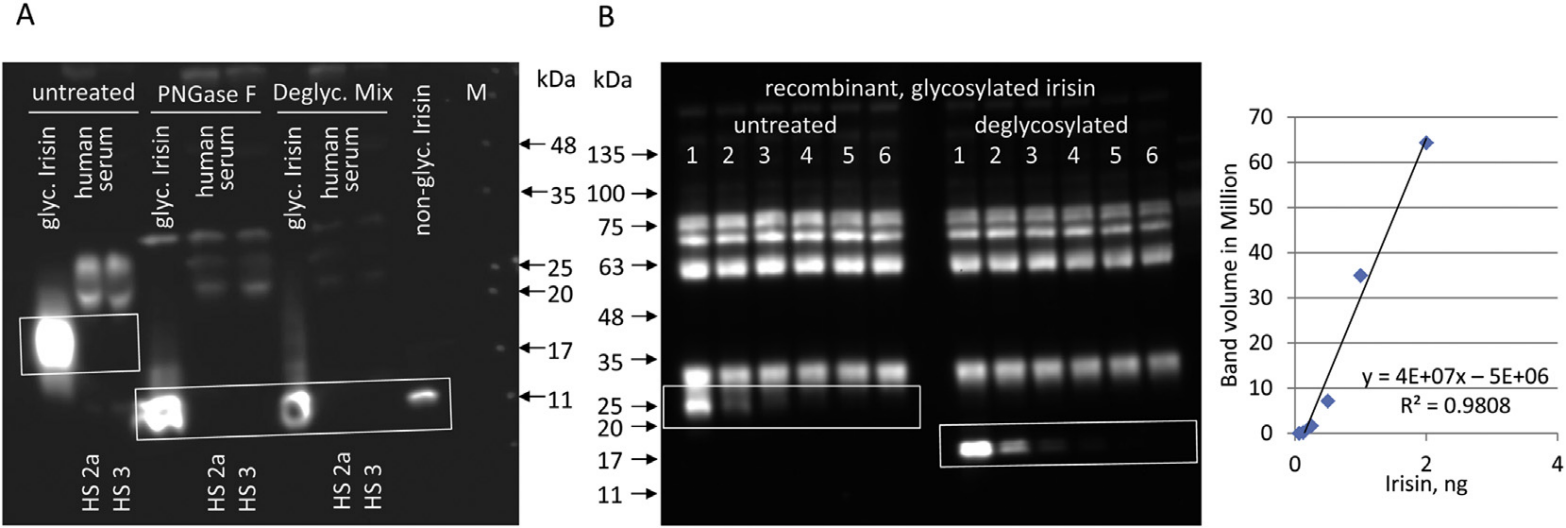
**Detection of irisin before and after deglycosylation. A:** Deglycosylation of recombinant glycosylated irisin and human serum samples* with PNGase F or protein deglycosylation Mix II (NEB) and detection with antibody C. *Human samples: HS 2a, HS 3 (acute exercise; details in Supplementary Table S1). **B:** Detection of different amounts of recombinant glycosylated irisin (2, 1, 0.5, 0.25, 0.125, and 0.062 ng) with antibody B before (lanes 1–6, left panel) and after deglycosylation (lanes 1–6, right panel). Glycosylated recombinant irisin was added to albumin-depleted bovine plasma. Linearity of detection is shown in the right panel. Glycosylated irisin (left panel) could not be quantified (details in Supplementary Figure S2D). M: molecular weight marker. Regions of interest are marked by boxes.

The efficiency of deglycosylation of recombinant human glycosylated irisin was tested with PNGase F or a deglycosylation mix (Protein Deglycosyation Mix II; NEB) in four independent experiments. We used the same antibody (antibody D) that Jedrychowski et al. [7] used to identify the bands in their mass spectrometry measurement of irisin. The samples were albumin- and IgG-depleted before deglycosylation. Deglycosylation of irisin with PNGase F was as effective as with the enzyme cocktail. Large amounts of human recombinant glycosylated irisin were apparently completely deglycosylated as shown in Figure 4A. No specific bands for irisin were detected in human serum samples incubated with either PNGase F or the deglycosylation mix (Figure 4A). Notably, the samples were from individuals who recently exercised.

Although no differences in the efficiency of deglycosylation of recombinant irisin between PNGase F and the Protein Deglycosylation Mix II (NEB) were observed, all subsequent experiments involving deglycosylation of the samples were conducted with the mix.

We then tested antibody B for its sensitivity and specificity for the detection of recombinant glycosylated irisin before and after deglycosylation (Figure 4B and Supplementary Figure S2D).

Glycosylated irisin was visible at higher concentrations but was too close to unspecific staining to be quantified by image analysis. After deglycosylation, an irisin band appeared at the expected MW of ∼13 kDa and was detected with similar sensitivity as previously described for this antibody (Supplementary Figure S2B).

### 3.5 No detection of circulating, native irisin by western blotting after resolution of high total protein amounts

**Figure 5:**
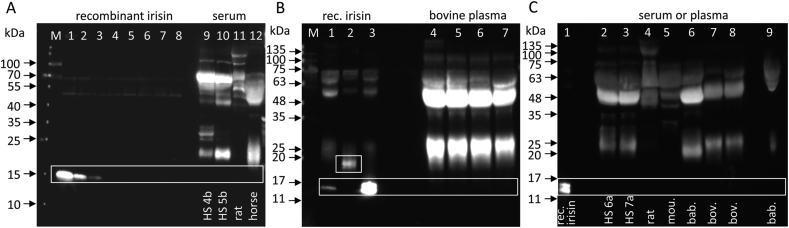
**Detection of recombinant and native irisin in different species with antibody B. A:** Recombinant irisin (lanes 1–8: 4, 2, 1, 0.5, 0.25, 0.125, 0.062, and 0.031 ng) and native irisin in sera of human*, rat, and horse detected with antibody C. All sera were albumin- and IgG-depleted, deglycosylated and 80 μg protein were resolved per lane. *Human samples: HS 4b and HS 5b (12 w training; details in Supplementary Table S2). **B:** Recombinant irisin (lane 1); recombinant glycosylated irisin before (lane 2) and after (lane 3) deglycosylation. Bovine plasma was spiked with 50, 100, 200, and 400 ng/ml irisin, albumin- and IgG-depleted, deglycosylated and 120 μg protein were resolved (lanes 4–7) and detected with antibody B. **C:** Human*, rat, mouse (mou.), baboon (bab.), and bovine (bov.) plasma/serum samples were albumin- and IgG-depleted and deglycosylated and 120 μg total protein were resolved per lane. Native baboon plasma (2 μL with ∼100 μg protein) was applied to lane 9. Lane 1: 20 ng deglycosylated recombinant irisin. Detection with antibody B. *Human samples: HS 6a and HS 7a (12 w training + acute exercise; details in Supplementary Table S1). M: molecular weight marker. Regions of interest are marked by boxes.

We increased the analyzed amounts from the standard 20–50 μg total protein per lane to 80–120 μg after albumin and IgG-depletion, sample concentration, and deglycosylation of serum/plasma from the different species. No specific bands were observed in the serum samples of any species. Notably, the humans were subjected to different exercise protocols prior to sampling. Instead, heavy staining of unspecific bands occurred in the tested irisin antibodies B (Figure 5B,C) and C (Figure 5A). Specific bands at 13 kDa appeared in the lanes with higher concentrations of recombinant irisin or deglycosylated recombinant irisin only. Recombinant glycosylated irisin was detected at the expected MW of ∼20 kDa. Of note, recombinant glycosylated irisin was spiked into the cattle samples (50, 100, 200, and 400 ng/ml) before sample processing but was not detected (Figure 5B).

Loading of 160 μg albumin- and IgG-depleted protein per lane led to massive unspecific staining by the antibody (data not shown).

### 3.6 Detection of FNDC5 and irisin in muscle and serum by targeted mass spectrometry: critical role of sample preparation

A multi-step procedure to remove albumin and IgG and concentration of the retained samples prior to deglycosylation was compared in human, rat, and horse serum samples (Table 2). Removal of IgG and albumin should result in ∼80% loss of the total proteins according to the manufacturer. The subsequent concentration step might cause additional protein losses of 5% in the filters’ selected molecular weight range.

**Table 2:** Characteristics of the serum samples before and after processing.

Serum samples^a^	Original sample		Sample after processing^b^			Batch	Subsequent analysis^c^
	Protein concentration (mg/mL)	Protein amount (mg/115 μL)	Retained volume (μL)	Retained protein			
				mg	(%)		
Human HS 2b	54.41	6.26	70	1.02	16.2	2	
Human HS 4a	58.80	6.76	80	1.12	16,6	3	
Human HS 4b	53.99	6.21	90	1.21	19.5	3	
Human HS 5a	65.51	7.53	75	1.74	23.1	3	
Human HS 5b	62.54	7.19	100	1.20	16.6	3	
Human HS 6b	51.94	5.97	75	1.73	29.0	1	
Human HS 6b (technical replicate)			105	1.23	20.6	1	MS (PRM)
Human HS 7b	56.19	6.46	95	1.23	19.1	1	MS (PRM)
Human HS 7b (technical replicate)			75	1.79	27.7	1	MS (PRM)
Human HS 7c	54.92	6.32	70	1.02	16.1	2	MS (DDA)
Rat	45.89	5.28	50	2.04	38.7	3	MS (DDA)
Rat (technical replicate)			70	2.28	43.2	1	MS (PRM)
Horse	54.71	6.29	100	1.34	21.3	3	

a See Supplemental Table S1 for detailed sample data.

b Removal of albumin and IgG with ProteoExtract (Calbiochem); concentration of proteins with Amicon Ultra 0.5 Centrifugal Filter Devices (Merck).

c MS: mass spectrometry; PRM: parallel reaction monitoring; DDA: data dependent acquisition.

The retained amounts of protein in the human samples after processing (16–29%) were in the expected range as described by the manufacturers. In the rat sample, 39% and 43% of total protein were retained in two separate preparations. There was a considerable variability between the samples processed in different batches and also between aliquots of the same sample processed in the same batch.

For targeted mass spectrometry, human and rat sera were deglycosylated after albumin-/IgG-depletion and concentration and 160 μg total protein was separated per sample (Supplementary Figure S3A). Untreated human muscle protein was analyzed as well as human and murine muscle protein after deglycosylation (50 μg per lane). Recombinant glycosylated irisin after deglycosylation was used as a control. The regions of interest are marked by boxes and are described in Supplementary Figure S3B.

To detect the peptide DSPSAPVNVTVR, which is not a regular tryptic fragment of FNDC5 and requires transcription from the non-canonical start codon as well as cleavage of the signal peptide, the irisin sequence was added to the sequence database. Neither specific peptide signatures were found in the negative control samples nor in the 5–10 and 20–30 kDa ranges removed from the serum samples. The tryptic irisin fragment (FIQEVNTTTR) was found in the rat but not in the human serum at 10–15 kDa. No sign of the fragment DSPSAPVNVTVR was detected in any molecular weight region of the serum samples (Supplementary Figure S3B).

Both peptides for FNDC5/irisin were found in the positive control (recombinant glycosylated irisin after deglycosylation) and in all of the muscle samples. As expected, the peptides in the deglycosylated samples showed deamidation of the respective asparagine residues (N > D) as a consequence of deglycosylation. Of note, a weak signal indicative of deamidation was also found in the untreated muscle samples in addition to a strong signal for the unmodified peptide.

### 3.7 Quantification of native irisin in deglycosylated human and rat serum by mass spectrometry

**Figure 6:**
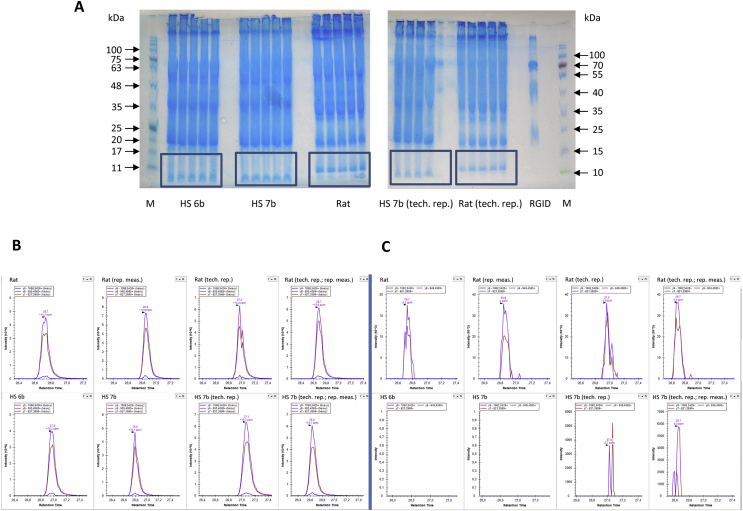
**Detection of irisin in rat and human serum by mass spectrometry (PRM mode). A:** Coomassie-stained gels with boxed regions of interest removed for targeted mass spectrometry in the PRM mode; 300 μg total protein per sample was subjected to electrophoresis (5 × 60 μg). Only ∼270 μg total protein was available for sample HS 7b. **B:** PRM elution profile for the heavy AQUA irisin peptide (FIQEDVTTTR) in the rat (upper panel) and human (lower panel) sera. **C:** Corresponding profiles of the tryptic irisin peptide (FIQEDVTTTR). *Human samples: HS 6b (12 w training) and HS 7b (12 w training + acute exercise; details in Supplementary Table S1).

We used the tryptic peptide (^48^FIQE**V**DTTTR^57^) labeled with a heavy valine residue (V) as standard for quantitative mass spectrometric analysis. This peptide contains an aspartate residue (D) instead of the original asparagine (N) accounting for the deamidation of asparagine after enzymatic deglycosylation. Two human and one rat serum samples were depleted of albumin and immunoglobulins, enzymatically deglycosylated, and 300 μg protein (5 lanes with 60 μg protein per sample) were resolved by denaturing polyacrylamide gel electrophoresis. Although none of the tested antibodies stained bands in the size range where deglycosylated native irisin would be expected (13 kDa), and even 400 ng/ml of recombinant glycosylated irisin dissolved in bovine plasma did not yield a visible band after sample processing (Figure 5B), the respective gel regions (10–15 kDa) were removed according to the positions of size markers (boxes in Figure 6A). A total of 18.5 fmol of the labeled peptide was added per 1 μL of the samples prior to quantification. The PRM elution profiles of the heavy peptides are shown in Figure 6B, and the results of the respective deamidated serum peptides are shown in Figure 6C. The upper parts of Figure 6B,C depict the data for a single rat sample from two subsequent serum preparations with repeated measurements.

In contrast, no traces of irisin-specific peptides were found in the two human serum samples (HS 6b and HS 7b, Figure 6C). However, repeated preparation and repeated quantification of sample HS 7b detected the deamidated peptide. Only 270 μg instead of 300 μg total protein were available for electrophoresis of this sample (Figure 6A). The fragmentation pattern and MS/MS spectrum of the analyzed peptide are shown in Supplementary Figure S4. Quantification of the peptides and the calculated irisin concentrations in the original samples are listed in Table 3.

**Table 3:** Quantification of irisin in human and rat serum.

Sample^a^	Remarks	Amount of peptide (^48^FIQEVDTTTR^57^; fmol)	Calculated irisin concentration^b^ (ng/mL serum)
HS 6b		N/D	–
HS 7b		N/D	–
HS 7b	Technical replicate	0.42	0.091
	Repeated measurement	0.70	0.153
Rat		2.84	0.618
	Repeated measurement	3.60	0.783
Rat	Technical replicate	4.20	0.914
	Repeated measurement	4.35	0.946

N/D: Not detected.

a See Supplemental Table S1 for detailed sample data.

b fmoles of peptide x 25 kDa (MW of glycosylated irisin) x 8.696 (for 115 μL processed serum).

The rat samples contained between 2.8 and 4.4 fmol of the peptide and the human samples contained 0.4 and 0.7 fmol in repeated measurements. The calculated irisin concentrations ranged from 620 to 950 pg/mL in the rat sample and from 90 to 150 pg/mL in the human sample (Table 3).

## 4 Discussion

Crosstalk between muscle and organs such as the liver, gut, pancreas, adipose tissue, bone, and brain may be mediated by secreted myokines (reviewed by Görgens et al. [31], Pedersen [32]). Irisin is considered a promising myokine linking physical activity to beneficial health effects. Recent studies reported positive effects of irisin on adipose tissue browning, bone structure, and cognitive ability [1,[10], [11], [12]]. Many studies measured circulating irisin after exercise, the relationships between irisin levels and pathological conditions, and the *in vitro* as well as *in vivo* effects of recombinant irisin on signaling pathways. However, there is still considerable heterogeneity in reports on circulating amounts of irisin in humans [7,19,33] and molecular weights of different forms of irisin and its precursor FNDC5 in humans and mice [1,[11], [12], [13]]. Thus, we addressed FNDC5 gene structure and the detection and quantification of FNDC5 and irisin.

To date, FNDC5 transcript abundance in human muscle has been assessed by only one experiment [30], and there are no data on putative FNDC5 protein variants. This is important because the expression of full-length human FNDC5 requires transcription from a non-canonical ATA start codon, whereas translation from a downstream canonical ATG leads to the expression of a truncated protein containing only parts of the proposed irisin peptide. The current study demonstrates that only one of three annotated human FNDC5 transcripts is expressed as predicted in human skeletal muscle. Moreover, at least two additional transcripts exist with different combinations of exons 5 and 6. All of the observed transcripts are likely to be translated from a non-canonical AUA start codon in exon 1b. Exons encoding the irisin peptide were expressed in all but one observed transcript, whereas annotated transcript 1 was not found. This is in line with our prior research [6] and supported by experimental data of Kim et al. [30] that identified a CpG island and a promotor upstream of transcripts 2 and 3 but not transcript 1. Thus, translation of human FNDC5 from a downstream canonical AUG start codon resulting in an N-terminally truncated FNDC5 isoform is unlikely and confirms the results of Jedrychowski et al. [7]. As a consequence, data on the expression of annotated transcripts 1 to 3 in human tissues [30] must be considered with caution because they monitored expression of different combinations of exons 4–6, which may not be representative of the annotated transcripts. FNDC5 transcript diversity in humans is similar to what we observed in cattle [25] and contrasts the expression of only a single transcript in murine muscle in our present study.

Specific FNDC5 bands were found by western blotting in murine, bovine, human muscle, and murine SAT samples but not in human and bovine SAT. Both human SAT samples were from healthy, normal weight individuals. This is concordant with an earlier report in which an FNDC5 antibody failed to mark a specific band in human SAT samples but stained a band in rat SAT at ∼25 kDa [34]. In another study, FNDC5 was detected in human visceral adipose tissue but only in SAT of obese individuals [35]. Both studies used antibodies raised against the C-terminus of FNDC5 similar to our present study (Table 1), and the tissue extracts were not deglycosylated. Collectively, the data suggest differences in FNDC5 expression in adipose tissues of rodents compared to other species including humans despite similar expression in skeletal muscle. Whether an expression of FNDC5 in human SAT is ectopic or related to obesity remains to be investigated. Thus, our results oppose the findings of Roca-Rivada et al. [34] and Moreno-Navarrete et al. [36] that also considered FNDC5 an adipokine.

Some issues regarding the apparent molecular weight of FNDC5 in different tissues remain to be addressed. Many studies detected FNDC5 in a molecular weight range from 22 to 30 kDa in untreated muscle of different species [25,26,34,37,38]. Our blocking experiments confirmed this in human and mouse muscle. The theoretical size of glycosylated FNDC5 is ∼27 kDa in both species and should be reduced to a sharper band of ∼20 kDa after enzymatic deglycosylation. This was not observed in our experiment, although the presence of deglycosylated peptides was confirmed in the 20–27 kDa size range by mass spectrometry. Lourenco et al. [11] identified four immune-reactive bands of FNDC5 in protein extracts from mouse hippocampus at molecular weights between 27 and 75 kDa with a commercial antibody and confirmed the presence of FNDC5 protein in all bands by mass spectrometry. This was interpreted as the occurrence of multimers and/or post-translationally modified FNDC5 forms in the hippocampus [11]. However, the detection of protein multimers should be largely reduced under denaturing electrophoresis conditions. Respective investigations in muscle samples are still required.

We tested one custom-made and three commercial irisin antibodies. The antibodies detected recombinant irisin at amounts as low as 125 pg per lane in western blotting. In contrast, no specific band was found in the sera of different species. Incomplete deglycosylation of irisin in human serum samples with PNGase F was considered a cause of the failure to detect native irisin in western blotting [7]. However, this approach ignored that the initial detection of irisin in human and murine samples was described after deglycosylation only with this specific enzyme by the same group [1]. Moreover, our results showed no difference in the efficiency of deglycosylation of recombinant glycosylated irisin by PNGase F compared to the enzyme mix proposed by Jedrychowski et al. [7]. This indicates an exclusive N-glycosylation of recombinant irisin produced in HEK293 cells. We did not observe a specific irisin band in the size range between 10 and 15 kDa in human serum after both deglycosylation and incubation with the same antibody used by Jedrychowski et al. [7] and other antibodies. Our results contradict the data of the latter group reporting bands at 25 kDa before and at 12 kDa after deglycosylation of human serum and resolution of 50 μg protein per lane for western blotting (Table 1). Even with 80 μg–120 μg proteins resolved per lane and using the most sensitive antibody from our previous tests, we again failed to detect specific irisin bands. There are only a few published studies on irisin detection in human plasma or serum by western blotting. In the initial study on the discovery of irisin [1] as well as in subsequent analyses of Wen et al. [39] and Lee et al. [12], an antibody raised against the C-terminus excluding the irisin domain was used (Table 1). Bands with a molecular weight of 32 kDa prior to deglycosylation were reduced by PNGase F-mediated deglycosylation to 20–24 kDa. Lee et al. [12] explained this result with the possibility that shivering causes the release of (extra-irisin) FNDC5 fragments into the circulation containing antibody-reactive peptides. In contrast, Löffler et al. [37] detected irisin at 25 kDa in human serum. The band corresponded to the proposed size of glycosylated irisin in humans and deglycosylation with an enzyme mix resulting in a shift to ∼17 kDa. However, this antibody was discontinued by the manufacturer. Since then, only Jedrychowski et al. [7] detected irisin at 25/13 kDa in human serum using western blotting, in contrast to our results (Table 1). According to our data, there was a discordance between the minimum amount of irisin required for detection (∼125 pg per lane) and the maximum of total protein that could be loaded onto the gel for electrophoresis (120 μg). Sample preparation including concentration and deglycosylation resulted in a theoretical amount of 25 pg irisin per 120 μg total proteins at an irisin concentration of 3–4 ng/mL in human plasma [7]. This did not account for any irisin losses during preparation and was far below the detection limit of the most sensitive irisin antibody tested using western blotting in our study. Kim et al. [10] reported a short half-life (1 h) of injected recombinant irisin in mice. If this half-life is also valid for native irisin in mice and other species, handling until serum preparation is important.

Multiple cross-reactions with other serum proteins were observed in this and previous studies [6,40]. Because many of the antibodies used for western blotting are also part of ELISAs, this has implications for the reliability of irisin concentrations measured with ELISA. Not surprisingly, significant differences in the levels of circulating irisin ranging from 40 pg/mL to 2.2 μg/mL were reported in healthy humans in early studies [35,[41], [42], [43]]. This prompted us and others to publish cautionary notes [6,15]. Unfortunately, the situation has changed little then since, with recently measured irisin levels still spanning orders of magnitude (50 pg/mL to more than 10 μg/mL [11,19,[44], [45], [46]]). Comparisons between values measured in the same samples with different ELISAs, or even with two lots of the same ELISA, demonstrated significant discrepancies [6,15,47]. Consequently, data on irisin in human plasma and serum should not be used unless the reliability of irisin antibodies in ELISAs can be proven.

Detection and quantification of irisin by mass spectrometry is considered the gold standard and should resolve controversies regarding the validity of ELISAs [48]. Nevertheless, only a few studies employed this method to detect or quantify irisin in human serum [6,7,12,13,49,50] or rodent tissue and serum [40,51]. Other than the two studies detecting a FNDC5/irisin signature in western blotting bands marked by C-terminal anti-FNDC5 [1,12], only Jedrychowski et al. [7] found FNDC5/irisin-specific peptides in western blotting of human serum after anti-irisin staining. In contrast, Barja-Fernandez et al. [40] failed to find FNDC5/irisin signatures in marked 25 kDa and 15 kDa bands, whereas we previously found these signatures at 25 kDa without bands marked by irisin antibodies [6]. We observed herein FNDC5/irisin signatures in rat but not human serum samples after deglycosylation at 10–15 kDa. Peng et al. [51] demonstrated the presence of FNDC5/irisin signatures in fractionated murine serum (10–50 kDa) using mass spectrometry.

An independent reproduction of the quantification of irisin in human samples demonstrated by Jedrychowski et al. [7] has not been published to date. Only one comparable experiment was conducted but failed to detect FNDC5/irisin in serum samples of six humans by mass spectrometry even by processing 1,250 μg of total protein per serum sample [49]. In contrast, these studies measured irisin concentrations in human cerebrospinal fluid (CSF) of the same individuals in a range between 0.26 and 1.86 ng/mL, indicating the measurement sensitivity. We initially did not find FNDC5/irisin signatures in two human serum samples obtained after different types of exercise. However, repeated preparations of one sample led to a positive result, with 0.09 ng irisin/mL serum and 0.15 ng/mL in repeated measurements. This value was 25- to 40-fold lower than the levels reported by Jedrychowski et al. [7] for sedentary individuals (3.62 ng/mL). Furthermore, we quantified plasma irisin in a rat sample to be 0.62–0.95 ng/mL using repeated preparations and measurements. This was 2-3-fold more than recently measured in mouse plasma (0.3 ng/mL [10]).

The quantification of low abundance serum/plasma proteins requires the removal of highly abundant albumin and immunoglobulins prior to electrophoresis and mass spectrometric analyses. We observed a very high variability of protein recovery during this procedure and the subsequent concentration of retained proteins. Successful detection of FNDC5/irisin signatures required relatively high amounts of retained protein that were not obtained for all of the samples even when processed in a single batch. This calls into question the reproducibility of irisin quantification via mass spectrometry with absolute quantification (AQUA) peptides. Moreover, repeated measurements of the same sample resulted in further variability of the calculated irisin serum concentrations. Thus, a cautious biological interpretation of small differences in irisin levels between sedentary and exercised subjects in a previous study (3.62 ng/mL vs 4.33 ng/mL, p = 0.041) is suggested as even the authors reported a wide range (10–30%) of typical protein losses during preparation [7]. This study measured samples of four sedentary and six exercised subjects with two heavy AQUA peptides characteristic of the N-terminal and internal parts of irisin. Comparing the given values for both peptides in femtomoles, the amount of internal peptide was significantly larger than that of N-terminal peptide (18.40 fm vs 16.06 fm, p = 0.032) in the six subjects after aerobic exercise. The significance of this difference was not discussed in contrast to that between both groups [7]. Although the quantification of irisin with AQUA peptides is a very sensitive method that does not rely on antibody binding, it is impaired by the necessity of multi-step preparation of serum/plasma samples prior to the measurement.

Taken together, our results indicate no expression of transcripts encoding truncated FNDC5 proteins in humans, which is in line with previous results [7]. Irisin can be qualitatively detected in some but not all deglycosylated rat and human serum proteins at the predicted molecular weight of ∼13 kDa via mass spectrometry but not using western blotting with the tested antibodies. In contrast, its precursor FNDC5 can be consistently detected in the expected range with antibodies raised against its C-terminus. The failure to detect deglycosylated FNDC5 with reduced size in our experiment remains unclear. Recent results indicated the existence of additional FNDC5 species of larger size in mouse brain [11].

Many studies of mice and cell lines have demonstrated the effects of irisin (overexpressed or recombinant) on signaling pathways related to bone remodeling, brain function, and metabolism. Although the effects in most of these studies were observed only at somewhat high concentrations (nM to mM), a recent study demonstrated the effects of irisin at pM levels on bone turnover [10]. However, the current study demonstrated that there is still no reliable quantification method available for irisin in serum or plasma samples. This is prerequisite for studies on irisin's effects *in vivo* and unspecific binding of available irisin antibodies. Comparably, a systematic study on estrogen receptor beta antibodies concluded that insufficient antibody validation may challenge an entire research field [52]. Furthermore, sample preparation methods introduce uncontrollable variations into the quantification of irisin via mass spectrometry with AQUA peptides. A recent study of parallel irisin measurements using ELISA was considered reliable [9], and quantitative mass spectrometry demonstrated a correlation as low as 0.4 between the two methods [50]. Thus, measuring circulating irisin remains challenging.
