# Supplementary material for: Irisin: Still chasing shadows
Source: Mol Metab. 2020 Jan 31;34:124–35. doi: 10.1016/j.molmet.2020.01.016 (PMC7033458; doi:10.1016/j.molmet.2020.01.016)
Supplement: Multimedia component 1 [file mmc1.pdf]

Irisin – still chasing shadows

Elke Albrecht [1], Lisa Schering [1], Friedrich Buck [2], Konrad Vlach [3*], Hans-Christof Schober [3], Christian A. Drevon [4], Steffen Maak [1#]

[1] Institute of Muscle Biology and Growth, Leibniz Institute for Farm Animal Biology (FBN) Dummerstorf, Germany

[2] Institute of Clinical Chemistry, University Medical Center Hamburg-Eppendorf, Hamburg, Germany

[3] Department of Internal Medicine I, Municipal Hospital Suedstadt Rostock, Rostock, Germany

[4] Department of Nutrition, Institute of Basic Medical Sciences, University of Oslo, Oslo, Norway.

* Present address: Medical Clinic III, Angiology, University Hospital Dresden, Germany

[#] Corresponding author: Leibniz Institute for Farm Animal Biology (FBN), Institute of Muscle Biology and Growth, Wilhelm-Stahl-Allee 2, 18196 Dummerstorf, Germany, Phone: +49 38208 68850, E-mail: maak@fbn-dummerstorf.de

Supplementary Table S 1. Characterization of human serum samples analyzed in this study.

Subject/ Samples	Use	Exercise* (Sampling time after exercise)	Irisin level (ELISA)**	
			Phoenix (ng/mL)	Adipogen (µg/mL)
HS 1	Fig. 3	acute exercise (120 min)	125.9	2.44
HS 2a	Fig. 4	acute exercise (120 min)	168.0	3.91
HS 2b	Tab. 2	combined training (0 min)	102.6	4.18
HS 3	Fig. 4	acute exercise (120 min)	163.6	2.55
HS 4a	Tab. 2	No exercise (baseline)	124.0	2.30
HS 4b	Fig. 5A	combined training (0 min)	114.5	1.96
HS 5a	Tab. 2	No exercise (baseline)	109.2	3.17
HS 5b	Fig. 5A, Tab. 2	combined training (0 min)	130.3	2.97
HS 6a	Fig. 5C	combined training + acute exercise (120 min)	99.9	3.18
HS 6b	Fig. 6	combined training (0 min)	105.3	3.16
HS 7a	Fig. 5C	combined training + acute exercise (120 min)	182.7	2.77
HS 7b	Fig. 6, Tab. 2	combined training + acute exercise (0 min)	236.8	2.96
HS 7c	Fig. S 3, Tab. 2	combined training (0 min)	188.9	2.96

* Acute exercise: A 45-min bicycle test at 70 % of VO_2max was performed.
Combined training: Combined strength and endurance training for 12 weeks, including two endurance bicycle sessions (60 min) and two whole body strength training sessions (60 min) per week (Norheim et al. [5]).

** Irisin ELISAs: Phoenix (Cat.-#: EK-067-52; Phoenix Pharmaceuticals, Burlingame, USA; Norheim et al. [5])
Adipogen (Cat-#: AG-45A-0046Y; Adipogen Life Sciences, Liestal, Switzerland; Albrecht et al. [6])

Supplementary Table S 2. Primers for cDNA-PCR used in this study.

Primer	T_a [°C]	Amplicon length (bp)	Sequence 5'-3'	GenBank Accession no.
HS_FNDC5_1_F<br>HS_FNDC5_1_R	57	670	GTC GAG GTC CCA GCT GAG<br>CTG TCG CCA TGC CAG TCC	NM_001171941.2
HS_FNDC5_2_F<br>HS_FNDC5_2_R	57	671	GAG CCA CCA TAC ACC CCG<br>TGA GGG CAA GCA CTG AAA AG	NM_153756.2
HS_FNDC5_3_F<br>HS_FNDC5_3_R	57	557	GAG CCA CCA TAC ACC CCG<br>GCA GTC ACG CTT CAA TGA TGT CA	NM_001171940.1
HS_FNDC5_4_F<br>HS_FNDC5_3_R	60	250	TAC ATA GTC CAC GTG CAG GC<br>GCA GTC ACG CTT CAA TGA TGT CA	NM_001171940.1
HS_FNDC5_5_F*<br>HS_FNDC5_4_R*	60	292	CCT CCA AGA ACA AAG ATG AGG<br>TAC CAG AGC ATG AGG CAC AG	NM_001171941.2
HS_FNDC5_5_F*<br>HS_FNDC5_5_R*	60	247	CCT CCA AGA ACA AAG ATG AGG<br>TTT CAT ATC TTG CTG CGG AGA	NM_153756.2
HS_FNDC5_5_F*<br>HS_FNDC5_6_R*	60	160	CCT CCA AGA ACA AAG ATG AGG<br>ACA GGC AGT CAC GCT TCA AT	NM_001171940.1
HS_FNDC5_6_F<br>HS_FNDC5_7_R	57/60	160	AGA AAA GAG AGA GAG AGG TGC T<br>CTC CAG AAC ATC CCA GCT CA	NM_001171941.2
HS_FNDC5_7_F<br>HS_FNDC5_8_R	57/60	218	GGC TCT TCT CCC AAA CGG<br>GGT CTT CCT CTC CAT CCC C	NM_001171941.2
HS_FNDC5_8_F<br>HS_FNDC5_9_R	57/60	177	CCC GGC CAA TTC TAG AGT CT<br>GCA CAT CCT TCT TCT GCT GG	NM_153756.2;<br>NM_001171940.1
HS_FNDC5_9_F<br>HS_FNDC5_10_R	57/60	239	TCC ACC TTG TTT CTC TGG CT<br>GGG GTG TAT GGT GGC TCC	NM_001171941.2
HS_FNDC5_7_F<br>HS_FNDC5_9_R	57	177	GGC TCT TCT CCC AAA CGG<br>GCA CAT CCT TCT TCT GCT GG	NM_001171941.2
HS_FNDC5_6_F<br>HS_FNDC5_11_R	57	306	AGA AAA GAG AGA GAG AGG TGC T<br>GCC TGC ACG TGG ACT ATG TA	NM_001171941.2
HS_FNDC5_8_F<br>HS_FNDC5_12_R	57	206	CCC GGC CAA TTC TAG AGT CT<br>GGC CGC AAA GTC ATA CTC TG	NM_153756.2
HS_FNDC5_10_F<br>HS_FNDC5_12_R	57	395	TTT CGG ATA GTC GTG GGA GG<br>GGC CGC AAA GTC ATA CTC TG	NM_001171941.2
HS_FNDC5_11_F<br>HS_FNDC5_13_R	57	366	GCT TTG GGA GTC AGA TCA GC<br>ATC CTC CAG AAC ATC CCA GC	NM_001171941.2
HS_FNDC5_12_F<br>HS_FNDC5_14_R	57	364	TGT TCA AAT CCT GGC TCT GC<br>GGC AAA TCC GAT GAC AAC CT	NM_001171941.2
MM_Fndc5_1a_F<br>MM_Fndc5_1a_R	53	250	GAG CCG ATA TGC AGG C<br>CTG ACC ACG GCA GAG TTG	XM_006503212.3
MM_Fndc5_1b_F<br>MM_Fndc5_1b_R	57	189	GTG GCT AGG CTG CGT CTG<br>TCC TGA ATG AAC CGG AGC AT	NM_027402.4
MM_Fndc5_2_F<br>MM_Fndc5_2_R	57	245	TGC CAT CTC TCA GCA GAA GA<br>TCC TCC CCA TCT CCT TCA TG	NM_027402.4
MM_Fndc5_3_F<br>MM_Fndc5_3_R	57	304	TGA AGG AGA TGG GGA GGA AC<br>GCA TGT TGG ACA ATC ACC GT	NM_027402.4

HS: Homo sapiens; MM: Mus musculus

* Primer pairs from Kim et al. [30]

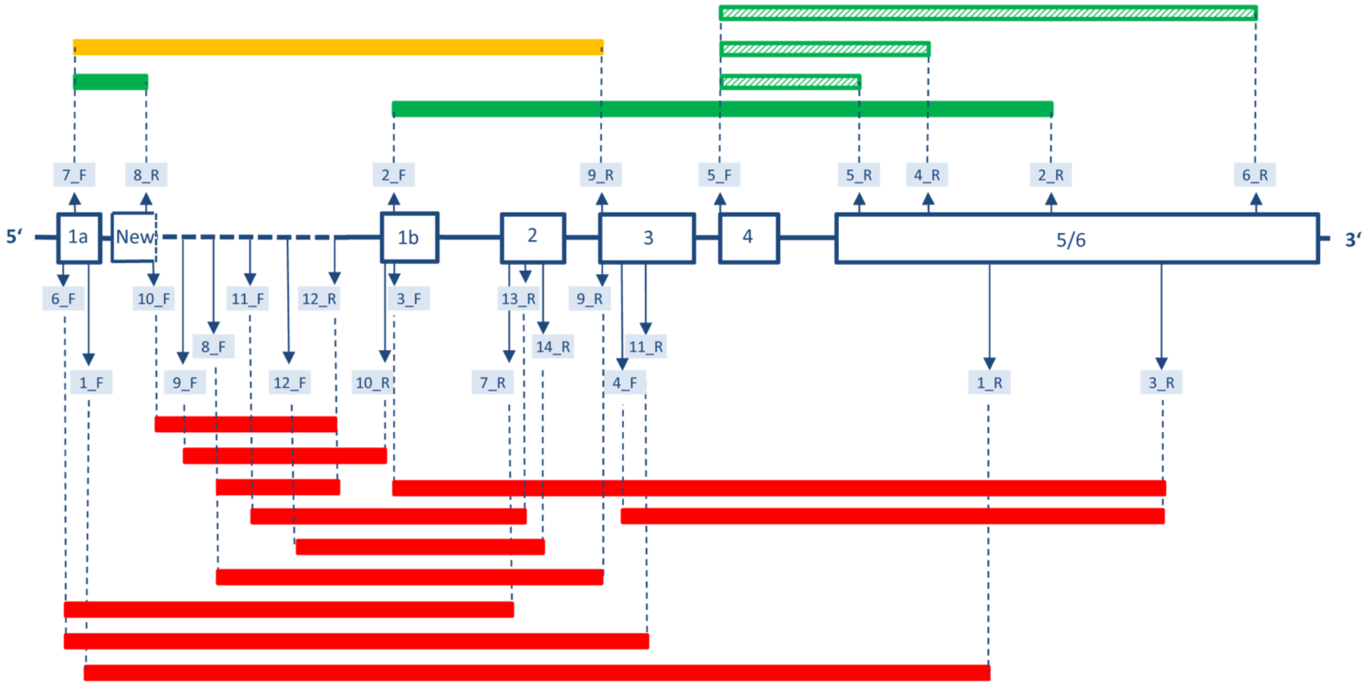

Supplementary Figure S 1. Primer combinations for *FNDC5* transcript analysis in human muscle.

The boxes refer to exons in the human *FNDC5* locus (compare Figure 1). The dashed line indicates a region annotated as intron but suspected to be expressed. Tested primer pairs are marked by arrows and the numbering relates to Supplementary Table S 2. Green bars: Successfully amplified and sequenced. Hatched green bars denote amplicons based on the primers published by Kim et al. [29]. Yellow bar: PCR product observed but no sequence derived. Red bars: Amplification failed.

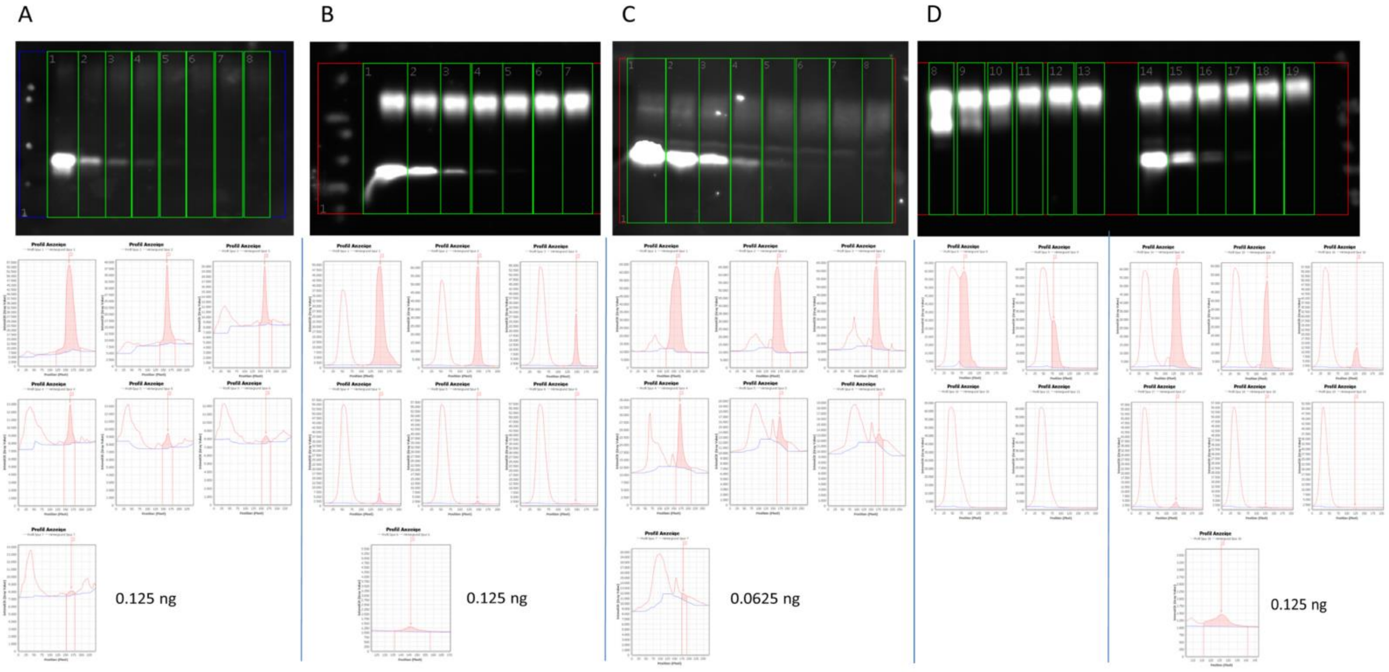

Supplementary Figure S 2. Image analysis of dilution series of recombinant irisin species on western blots.

A Recombinant irisin diluted in albumin-depleted bovine plasma. Irisin amounts per lane: 20, 4, 2, 1, 0.5, 0.25, 0.125, and 0.062 ng. Detection with antibody A. **B** Irisin amounts per lane: 4, 2, 1, 0.5, 0.25, 0.125, and 0.062 ng. Detection with antibody B. **C** Irisin amounts per lane: 4, 2, 1, 0.5, 0.25, 0.125, 0.062, and 0.031 ng. Detection with antibody C. **D** Glycosylated irisin amounts per lane: 2, 1, 0.5, 0.25, 0.125, and 0.062 ng (left panel). Same irisin amounts after deglycosylation with Protein Deglycosylation Mix II (NEB; right panel).

A

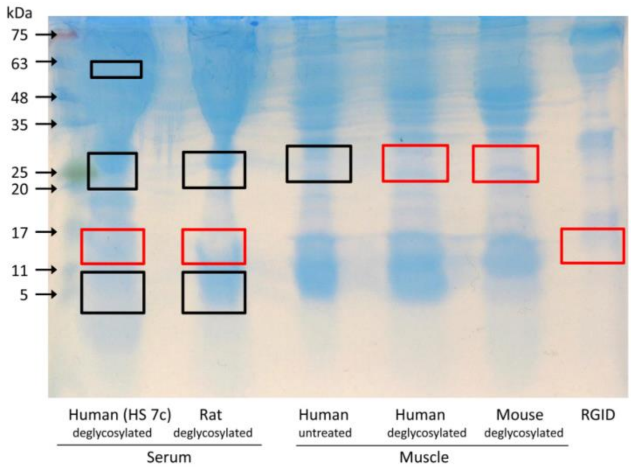

B

Sample	Molecular weight range (expected FNDC5/irisin form)	FNDC5/irisin signature
Human serum, deglycosylated (HS 7c*)	60 kDa (negative control)	-
	5 - 10 kDa (truncated irisin, T1)	-
	20 - 25 kDa (glycosylated irisin)	-
	10 - 15 kDa (deglycosylated irisin)	-
Rat serum, deglycosylated	5 - 10 kDa (negative control)	-
	20 - 25 kDa (glycosylated irisin)	-
	10 - 15 kDa (deglycosylated irisin)	FIQEVDTTTR
Human muscle, untreated	20 - 25 kDa (glycosylated FNDC5)	DSPSAPVDVTVR<br>FIQEVNTTTR
Human muscle, deglycosylated	20 - 25 kDa (deglycosylated FNDC5)	DSPSAPVDVTVR<br>FIQEVDTTTR
Mouse muscle, deglycosylated	20 - 25 kDa (deglycosylated Fndc5)	DSPSAPVDVTVR<br>FIQEVDTTTR
Recombinant, glycosylated irisin, deglycosylated (RGID)	10 - 15 kDa (deglycosylated irisin)	DSPSAPVDVTVR<br>FIQEVDTTTR

* HS 7c (12 w training; see Supplementary Table S 1 for details)

Supplementary Figure S 3. Detection of FNDC5/irisin by targeted mass spectrometry (DDA mode).

A Coomassie-stained gel with boxed regions of interest cut-out for targeted mass spectrometry in data dependent acquisition (DDA) mode. Red boxes sign areas where FNDC5/irisin was expected.

B Results of mass spectrometry with the detected peptides given in the last column.

Predicted Fragmentation Pattern

#1	b^+	b^{2+}	Seq.	y^+	y^{2+}	#2
1	148.07569	74.54148	F			10
2	261.15975	131.08352	I	1062.54258	531.77493	9
3	389.21833	195.11280	Q	949.45851	475.23290	8
4	518.26092	259.63410	E	821.39994	411.20361	7
5	617.32934	309.16831	V	692.35734	346.68231	6
6	732.35628	366.68178	D	593.28893	297.14810	5
7	833.40396	417.20562	T	478.26199	239.63463	4
8	934.45164	467.72946	T	377.21431	189.11079	3
9	1035.49932	518.25330	T	276.16663	138.58695	2
10			R	175.11895	88.06311	1

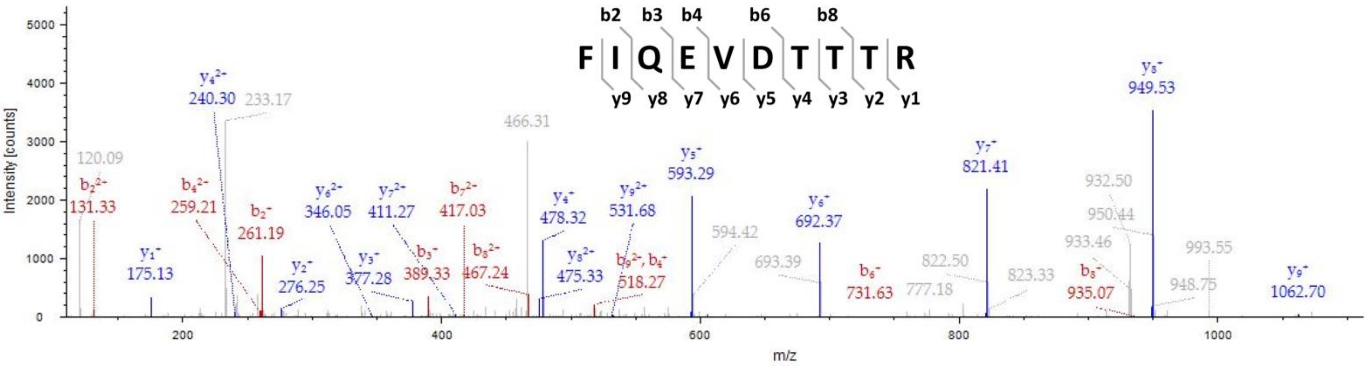

Supplementary Figure S 4. MS/MS spectrum of the deglycosylated, recombinant irisin peptide with b- and y-ion series m/z values.
